# Evaluation of semiochemical based push-pull strategy for population suppression of ambrosia beetle vectors of laurel wilt disease in avocado

**DOI:** 10.1038/s41598-020-59569-0

**Published:** 2020-02-14

**Authors:** Monique J. Rivera, Xavier Martini, Derrick Conover, Agenor Mafra-Neto, Daniel Carrillo, Lukasz L. Stelinski

**Affiliations:** 1 Department of Entomology, University of California Riverside, Riverside, CA USA; 2 Department of Entomology and Nematology, North Florida Research and Education Center, University of Florida, Quincy, FL USA; 3 ISCA Technologies, Inc., Riverside, CA USA; 4 Department of Entomology, Tropical Research & Education Center, University of Florida, Homestead, FL USA; 5 Department of Entomology and Nematology, Citrus Research and Education Center, University of Florida, Lake Alfred, FL USA

**Keywords:** Behavioural ecology, Agroecology

## Abstract

Ambrosia beetles (Coleoptera: Curculionidae: Scolytinae and Platypodinae) bore into tree xylem to complete their life cycle, feeding on symbiotic fungi. Ambrosia beetles are a threat to avocado where they have been found to vector a symbiotic fungus, *Raffaelea lauricola*, the causal agent of the laurel wilt disease. We assessed the repellency of methyl salicylate and verbenone to two putative laurel wilt vectors in avocado, *Xyleborus volvulus* (Fabricius) and *Xyleborus bispinatus* (Eichhoff), under laboratory conditions. Then, we tested the same two chemicals released from SPLAT flowable matrix with and without low-dose ethanol dispensers for manipulation of ambrosia beetle populations occurring in commercial avocado. The potential active space of repellents was assessed by quantifying beetle catch on traps placed ‘close’ (~5–10 cm) and ‘far’ (~1–1.5 m) away from repellent dispensers. Ambrosia beetles collected on traps associated with all in-field treatments were identified to species to assess beetle diversity and community variation. *Xyleborus volvulus* was not repelled by methyl salicylate (MeSA) or verbenone in laboratory assays, while *X. bispinatus* was repelled by MeSA but not verbenone. Ambrosia beetle trap catches were reduced in the field more when plots were treated with verbenone dispensers (SPLAT) co-deployed with low-dose ethanol dispensers than when treated with verbenone alone. Beetle diversity was highest on traps deployed with low-dose ethanol lures. The repellent treatments and ethanol lures significantly altered the species composition of beetles captured in experiment plots. Our results indicate that verbenone co-deployed with ethanol lures holds potential for manipulating ambrosia beetle vectors via push-pull management in avocado. This tactic could discourage immigration and/or population establishment of ambrosia beetles in commercial avocado and function as an additional tool for management programs of laurel wilt.

## Introduction

Ambrosia beetles (Coleoptera: Curculionidae: Scolytinae and Platypodinae) bore into trees to complete their life cycle within xylem galleries. However, while ambrosia beetles damage the tree during entry, they do not directly feed on the plant tissue; they have obligate nutritional relationships with symbiotic fungi^1^. The fungi are introduced to the tree during initial tree colonization and then, the beetles feed and reproduce while consuming them. Although neither ambrosia fungi nor beetles are monophyletic, both groups are named so because of their close association^2^. Once the fungi are established in the tree, the beetles begin to deposit eggs. Their mating systems vary greatly from haplodiploid scolytine species of the tribe *Xyleborini* to harem polygyny in *Pityophthorus lautus* (Eichhoff; tribe Corthylini)^3^. Economically, ambrosia beetles do the most damage when their symbiotic fungus is also a plant pathogen. This is the case with the redbay ambrosia beetle, *Xyleborus glabratus* (Eichhoff), which carries the causal agent of laurel wilt disease in Laureacae, *Raffaelea lauricola*^4^.

The varying life history strategies of ambrosia beetles may influence the spread of the symbiotic, plant-pathogenic fungi. Three general strategies have been used to classify this variation: primary, secondary, and saprophytic^5^. Primary bark beetles attack healthy trees; secondary infest weakened, stressed or recently killed trees; and saprophytic colonize dead host trees. Often, the three strategies overlap as the tree progresses towards death. Among the strategies, saprophytes are most common. Initially, it was thought there were tight mutualistic relationships between beetle species and the fungi with which they associate^6^. Recent research has shown the promiscuous nature of the association between ambrosia beetles and ambrosia fungi indicating movement of fungal species among multiple beetle species^7–9^.

The redbay ambrosia beetle, *Xyleborus glabratus*, is generally characterized by the primary life history strategy and attacks healthy trees^4^; its symbiotic fungus exhibits primary pathogenicity and is lethal to host trees without the vector^10^. *X. glabratus* was first found in the United States in 2002 near Savannah, GA^11^. Shortly after its discovery, it was linked to reports of dead and dying redbay trees (*Persea borbonia* L.). Laurel wilt has killed millions of native Lauraceae throughout the southeastern United States^12^. Although the disease is currently localized within the southeastern US, it also poses a threat to native Lauraecae on the west coast^13^, as well as, to the avocado industry.

In the United States, avocado is an important regional crop in Miami-Dade County Florida and southern California in counties such as Riverside, San Diego, Santa Barbara and Ventura. The production value of fresh market avocados was 390 million dollars in 2017 with over 20,200 ha harvested in California and over 2,000 ha harvested in Florida^14^. Laurel wilt is currently a serious problem in Florida’s avocado acreage and has caused significant yield decline since its discovery^14–16^. Florida production is primarily of West Indian (*Persea americana* var. *americana*) and Guatemalan (*P. americana* var. *guatemalensis*) avocado^17^, while California primarily produces the ‘Hass’ (*P. americana* var. *drymifolia*, a hybrid of Guatemalan and Mexican genomes) with 7–10 lesser grown varieties regionally^18^. Initially, laurel wilt causes wilting of terminal leaves that rapidly die and fall off the tree in 2–3 months after symptoms appear^19^. Internally, the wood turns color from a red-toned brown to a blue toned grey as the tree dies^19^. All of the most important varieties expressed differential severity of disease symptoms when inoculated; West Indian cultivars are the most susceptible^19^. *Xyleborus glabratu*s females bore into many of the most important avocado varieties including Hass, but symptomatic infection is dependent on variety^20^. However, avocado is not a highly attractive host of *X. glabratus*^21^ and this beetle species is rarely detected within avocado in Florida ambrosia beetle surveys^22,23^.

While *X. glabratus* is largely undetected in Florida avocado^22^, the promiscuous association between beetles and the ambrosia fungi suggests that other species also carry *R. lauricola*. *Raffaelea lauricola* is associated with multiple other beetle species reared from laurel wilt infected trees and some of those species are found consistently in avocado and are capable of transmitting *R. lauricola* to avocado^7,22^. In particular, xyleborine ambrosia beetles are compelling potential vectors because of their haplodiploid reproduction system. With this strategy, foundress females release and propagate their fungal symbionts in natal galleries within tree xylem. Females carrying the saprophytic or pathogenic fungi may be attracted to stressed trees with established fungal symbionts which could potentially contribute to the lateral spread of pathogenic fungi to new generations^24^. Males are flightless and develop from unfertilized eggs, which implies that a single female can begin a full brood within trees without the effort of finding a mate^25^. Multiple xyleborine species have been confirmed carriers of *R. lauricola* including *Xyleborus bispinatus* (Eichhoff), which is now considered a putative vector of laurel wilt in avocado^9,26^.

The objective of the present research was to investigate the possible application of volatile repellents and attractants as a push-pull tactic to reduce ambrosia beetle populations in avocado. The volatile organic compounds (VOCs) tested as repellents were verbenone and methyl salicylate (MeSA), which were previously shown to effectively repel *X. glabratus* in natural forest settings^27^. Experiments were initiated in the laboratory to determine if these volatiles had broad-spectrum effects on ambrosia beetle species beyond *X. glabratus*. We focused on *Xyleborus volvulus* and *X. bispinatus*, two species with known promiscuous association with *R. lauricola*^7,9^. Subsequent field testing evaluated repellents released from the SPLAT matrix, a medium that slowly releases volatiles over time, and that could be easily applied to the avocado trees. In the field, we tested both repellants with and without an attractant employed as a ‘pull’. The attractant was a low dose ethanol^24,28,29^. Treatment efficacy was assessed by measuring capture of naturally occurring ambrosia beetle species in test plots with unbaited traps. Recently, Byers *et al*.^30^ demonstrated that the active space of a repellent blend of piperitone and verbenone inhibited attraction of polyphagous shot hole borer, *Euwallacea fornicatus* (Shedl)^31^, to the female attractant, quercivorol, but only when repellent and attractant were separated by <1 m. These results indicated short-range behavioral activity (<1 m) of field-deployed monoterpene ketones against *E. fornicatus*^30^. Therefore, we assessed beetle catch with monitoring traps deployed ‘close’ (~5–10 cm) and ‘far’ (~1 m) from verbenone dispensers.

## Materials and Methods

### Insects, chemicals, and laboratory olfactometer bioassays

*Xyleborus volvulus* and *X. bispinatus* were obtained from laboratory cultures reared according to previously described methods^32,33^. Briefly, beetles were reared on media created from avocado sawdust inoculated with *R. lauricola*. Media and beetles were contained in plastic tubes kept in a temperature controlled rearing room (25 ± 1 °C, 75% RH) in complete darkness^32,33^.

Verbenone (93% purity), MeSA (99% purity) and dichloromethane (99% purity) were obtained from Sigma Aldrich (St. Louis, MO). Sources of odor for release in behavioral bioassays were prepared according to the methods described in Martini *et al*.^34^. Test compounds were dissolved in dichloromethane to the correct dosage rate (0.1 or 1.0 μg/μl) and pipetted onto 2 cm Richmond cotton wicks (Petty John Packaging, Inc. Concord, NC, USA). The cotton wicks contained either 10 or 100 μg of the compound and were placed randomly among the four arms of an olfactometer described below creating two treatment arms and two control arms. Before placing the wicks in the arms, the cotton wicks were left for 15 min in a fume hood for solvent evaporation.

A four-choice olfactometer was used to test the repellency of the two beetle species to verbenone and MeSA in the laboratory. Dichloromethane was used as the carrier solvent for the treatments and used as the control (100 μL). The four-choice olfactometer used in this study was previously described in Martini *et al*.^35^. Briefly, the four choice olfactometer has four arms that depart from a 30 × 30 cm polytetrafluoroethylene (PTFE) square arena. Four independent odor fields were created in the chamber by a constant airflow of 0.35 l/min pushed through each arm of the olfactometer and by pulling air (0.50 l/min) (as shown in Martini et al. 2015) through the floor’s central air evacuation hole. The olfactometer floor and arms were covered with filter paper (25 cm diameter laboratory filter paper, Curtin Matheson Scientific, Houston, TX) to assist beetle movement. The filter paper was changed between each bioassay and the olfactometer was washed with acetone and Sparkleen detergent (Fisherbrand, Pittsburgh, PA) to remove traces of the treatment chemicals. Each arm of the olfactometer was connected to a 350 ml glass vial the collected the beetles as they moved toward the treatments and then to a custom-made air delivery system (ARS, Gainesville, FL). The ambient air supply was purified through a charcoal filtration system. A flowmeter was used to measure airflow (Varian, Walnut Creek, CA) to ensure equivalent airflow within each arm. The olfactometer was centered under a 150W high-pressure sodium grow light (Hydrofarm, Petaluma, CA). For each species tested, 25 adult female beetles were released between 16:00 and 17:00 hr in the center of the olfactometer. After 16 hours, the number of beetles in each of the four chambers was counted and recorded. The experiment was run six times for each treatment and species combination.

### Field trials

A 10-acre avocado orchard was selected in Homestead, FL (25.509735, −80.521506) based on known incidence of laurel wilt disease symptoms. Avocado trees (West Indian) were 11 years old and planted on a 3 × 6 m spacing with average canopy height of 4 m. This orchard was adjacent to a second neighboring orchard approximately 10 m away and separated by a dirt roadway. The experimental orchard was managed through fungicide treatments and symptomatic tree removal and had low incidence of disease symptoms during the study while the neighboring orchard was characterized by visibly symptomatic trees and unknown management strategies. The experiment was deployed on the exposed edge of the managed orchard across from the orchard with disease symptoms and unknown management. We chose this area for investigation of chemical repellants and/or push-pull management based on the assumption that an orchard border facing a large area of marginally protected avocado should encourage high pressure from target beetles. The SPLAT (ISCA Technologies, Riverside, CA, USA) flowable matrix was used in the field as the release device for putative repellant chemicals. SPLAT containing either 10% w/v of verbenone or MeSA was dispensed as eight individual 1 g dollops per tree using a caulk gun (HDX, Heavy Duty Caulk Gun, Home Depot, Cobb County, GA, USA) to all trees per 3 × 3 tree plot. The application dosage was based on field efficacy observed previously with release of both verbenone and MeSA in previous field studies with *X. glabratus*^27^.

The initial field trial was established 4 May 2018 (Supplementary Figure 1). The objectives of the first experiment were: (1) to evaluate verbenone and MeSA as possible repellents for ambrosia beetle species occurring under field conditions in avocado, and (2) to determine whether active space of repellent dispensers was beyond 1 m in distance. Treatments were applied in to the 3 × 3 plots (0.05 ha) in a randomized complete block design with three treatments per block: verbenone, MeSA, untreated control and five replicates per treatment. Replicate blocks were separated by at least 20 m and treatment plots by at least 15 m. Four white, unbaited sticky traps were placed in each replicate block (Wing Trap Liners IPM-103, Great Lakes IPM, Vestaburg, MI, USA) with two traps on each of two trees, 1.5M above the ground. In each tree, two traps were stapled (top and bottom of the trap) onto a tree 1 m apart and at the same height. One trap was placed ~5–10 cm away from SPLAT dispensers (termed ‘close’), while the other was placed ~1–1.5 m away from dispensers (termed ‘far’). Traps were collected and replaced in the field every ten days for 30 days; traps were collected 10 days after treatment (DAT), 20 DAT, and 30 DAT which resulted in three sets of traps for the duration of the experiment. All ambrosia beetles caught on the sticky traps were counted, regardless of species.

The second field trial was established 12 June 2018 (Supplementary Figure 2). The objective of this experiment was to test the hypothesis that combining the forces of push (repellent, SPLAT with verbenone or methyl salycilate) and pull (attractant, an ethanol lure) would have a greater effect of inhibiting beetle populations as indirectly measured by capture on unbaited sentinel traps than deploying repellent alone. The same distribution of traps per plot was used; on each of two trees, two traps were placed with one “close” (~5–10 cm) and one “far” (~1.5 m) away from repellent. The attractant (‘pull’ factor) was ethanol dispensers (Product Number 3344, Synergy Semiochemicals, Burnaby, BC, Canada). The ethanol dispensers mimic stressed and declining trees and are attractive to a broad range of species of ambrosia beetles in avocado (see Results). The trial was deployed in the avocado orchard described above. The experiment was arranged as a split plot design replicated three times with the ‘pull’ as whole plot factor at two levels: (1) control, (2) ethanol dispensers and ‘push’ as the split plot factor at three levels: (1) control, (2) MeSA, and (3) verbenone. Like the first trial, SPLAT was applied to each tree in a 9 tree square plot (3 trees × 3 trees) with a buffer row in-between each plot. In the block with the ‘pull’ ethanol dispensers, the dispensers were put in the buffer row in-between SPLAT treatment plots. The ‘pull’ whole plot factor was established by deploying two ethanol dispensers per west facing plot edge along with a white sticky trap as described above to monitor beetle populations surrounding the ‘pull’ treatment. Control plots received unbaited sticky traps deployed without lures at west facing edge. This edge of plot borders faced the unmanaged avocado orchard described above. Beetle densities in each plot were measured with white, unbaited sticky traps placed by ‘close’ and ‘far’ away from nearest SPLAT dispenser, as described above. All replicate whole plots were separated by at least 20 m and split plots by at least 15 m. All traps were collected and replaced in the field every ten days. All Scolitydae beetles were identified to species level with the use of the most recent taxonomic key^33^. Voucher specimens are kept in the laboratory of Dr. Xavier Martini at the North Florida Research and Education Center in Quincy, FL, USA. Due to logistic constraints, beetles were identified at the species level only at 20 and 30 days after treatment (DAT).

### Statistical analyses

Beetle preference measured using the four-choice olfactometer was analyzed using a two-tailed, paired *t*-test in GraphPad Prism 7.05 (GraphPad Software, La Jolla, CA, USA). Field results were analyzed using a Generalized Linear Model (Proc GLM) to perform a repeated measures analysis of variance in SAS 9.4 (SAS, Cary, NC, USA). For the first field trial, the fixed variables in the model were: days after treatment (DAT), block, treatment, and trap distance. For the push-pull experiment, the fixed variables were: DAT, block, push (repellent), pull (attractant), and trap distance. A priori contrasts were applied to determine which treatment differed when significant difference were found. Analyses of community composition were performed with the statistical software R v 3.3.1 (R core team, Vienna, Austria). Diversity of Scolytineae beetles was examined with a principal component analysis (PCA) conducted with the package FactoMineR^36^. Linear correlation between principal components and beetle density were subsequently conducted.

## Results

### Laboratory olfactometer bioassays

**Figure 1:**
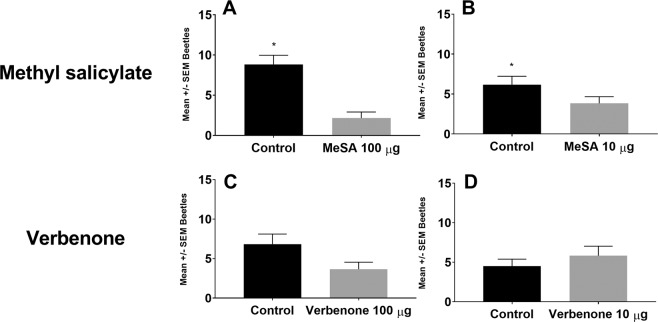
Response of *Xyleborus bispinatus* to methyl salicylate or verbenone at two loading dosages versus untreated clean air control. Interior graphs show response to methyl salicylate at two rates: 100 μg (**A**) and 10 μg (**B**) and verbenone at two rates: 100 μg (**C**) and 10 μg (**D**). Bars and error bars denote mean number of respondents and standard error respectively. Mean (±SEM) number of beetles without an asterisk did not differ significantly (α = 0.05).

**Figure 2:**
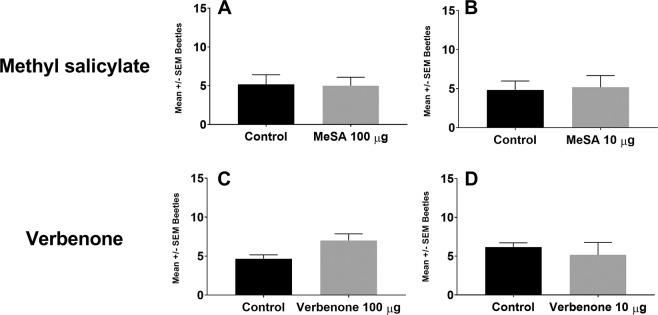
Response of *Xyleborus volvulus* to methyl salicylate or verbenone at two loading dosages versus untreated clean air control. Interior graphs show response to methyl salicylate at two rates: 100 μg (**A**) and 10 μg (**B**) and verbenone at two rates: 100 μg (**C**) and 10 μg (**D**). Bars and error bars denote mean number of respondents and standard error respectively. Mean (±SEM) number of beetles without an asterisk did not differ significantly (α = 0.05).

*Xyleborus bispinatus* was repelled by MeSA compared with the clean air control, at both 10 (t = 3.05, df = 5, P = 0.0284) and 100 μg (t = 3.964, df = 5, P = 0.0107) dosages, but was not significantly repelled by verbenone at either dosage (Fig. 1). At the higher 100 μg dosage of verbenone, there was a trend towards repellency (t = 2.109, df = 5, P = 0.0887, Fig. 1) for *X. bispinatus*. *X. volvulus* was not repelled by either MeSA or verbenone at either of the doses, as compared with the clean air control (Fig. 2).

### Field trial: repellents only

**Figure 3:**
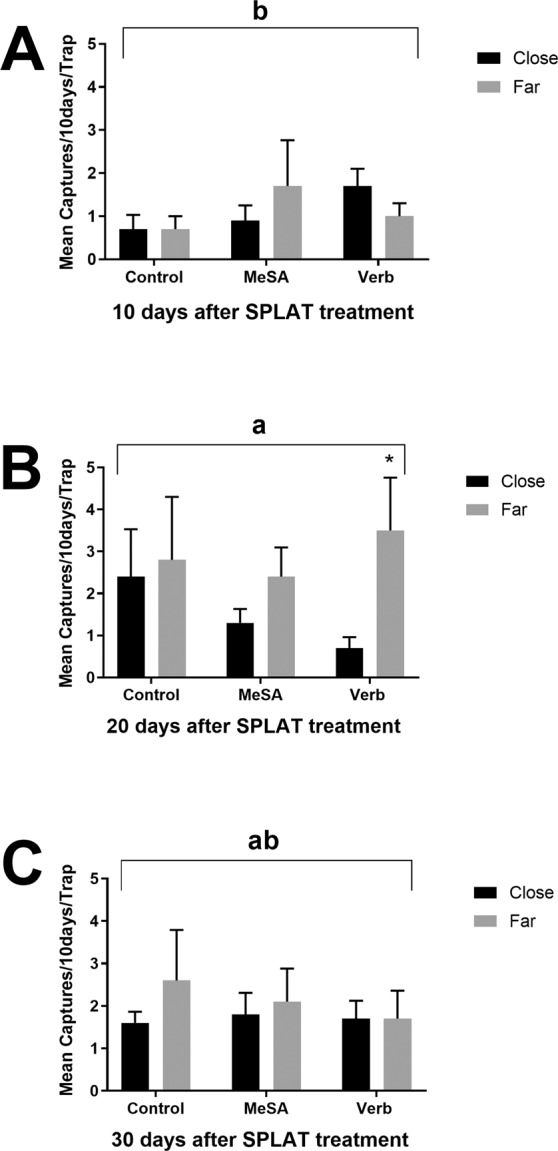
Effect of field-applied repellants by days after treatment and trap distance from dispensers on scolytine beetle trap captures without ethanol lures. Mean (±SEM) number of beetles without an asterisk did not differ significantly from its paired mean (α = 0.05). Traps were collected at ten day intervals with panel A representing captures from 0–10 days after application, panel B representing captures from 10–20 days after application, and panel C representing captures from 20–30 days after application. ‘Close’ indicates monitoring traps were ~5–10 cm from repellent dispenser, while ‘far’ indicates traps were ~1–1.5 m from repellent.

In the first field study (repellent only), days after treatment and sentinel trap distance from dispensers impacted number of beetles caught. There was no overall effect of repellent plot treatment on numbers of beetles caught on the associated monitoring traps. However, a trend for treatment effect was observed for reduced captures on sentinel traps deployed close to dispensers (F_1_ = 3.37, P = 0.0682). DAT influenced variation in trap captures; the number of beetles captured 20 DAT was significantly higher than captures at 10 or 30 DAT (F_2_ = 3.45, P = 0.0340; Fig. 3). DAT also had an effect on number of beetles captured in treatment plots by trap distance. At ten days (Fig. 3A) and thirty days (Fig. 3C), there was no statistical difference between captures on close or far traps in any of the plot treatments. However, when analyzed within the treatment times, at 20 DAT, fewer beetles were captured on traps placed “close” to verbenone dispensers than on control traps (F_1_ = 4.75, P = 0.0429; Fig. 3B). There was a similar trend observed at twenty days with reduced catches on traps located “close” to MeSA dispensers than on control traps or traps placed “far” from these dispensers (Fig. 3B). Overall, beetle catch on traps placed far away from either MeSA or verbenone did not differ from the controls.

### Field trial: complete push-pull system

**Figure 4:**
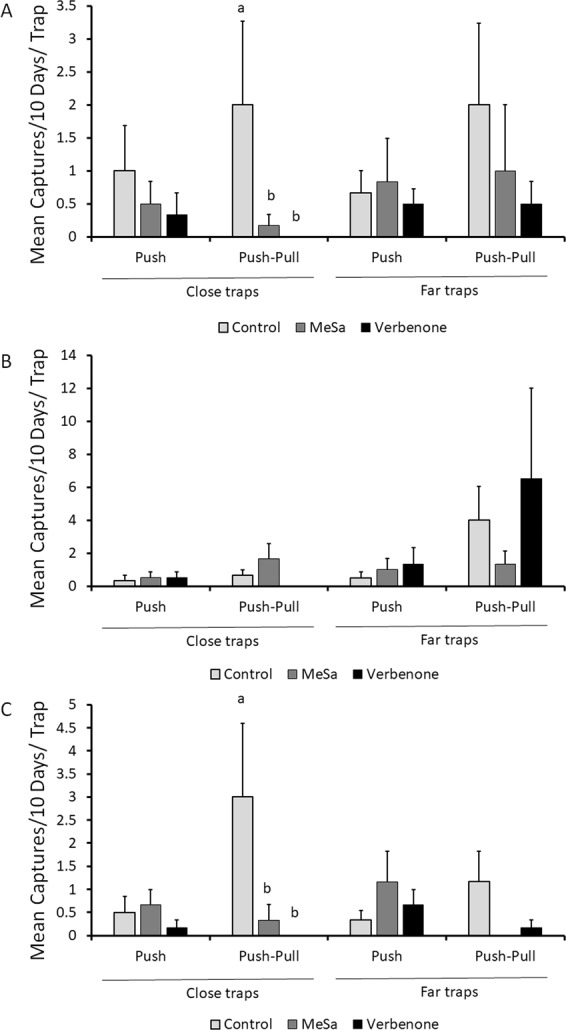
Effect of low-dose ethanol lures on ambrosia beetle trap captures in presence of repellents on mean (±SEM) Scolytinae beetle trap captures (**A**) from 0–10 days after treatments (DAT), (**B**) from 10–20 DAT, and (**C**) from 2030 DAT. Different letters indicate significant differences between treatment means. ‘Close’ indicates monitoring traps were ~5–10 cm from repellent dispenser, while ‘far’ indicates traps were ~1–1.5 m from repellent.

In the second field experiment, an ethanol dispenser (attractive pull) sub-factor was included in the design. We observed a significant effect of the interaction between the push (repellents in SPLAT) and the pull (ethanol attractant) treatments on beetles captured per trap deployed near treated trees (Table 1). The numbers of beetles captured in plots treated with the push and pull were significantly lower than in control plots at 10 (Fig. 4A) and 30 DAT (Fig. 4C). This was true for both push-pull using verbenone of MeSA as the repellent. Interestingly, not a single beetle was captured in close traps placed in plots treated with verbenone in the push-pull blocks throughout the entire experiment. Trap captures on the “close” traps did not vary significantly with sampling date while those on the “far” traps varied significantly by sampling date (Table 1). There was also an interaction between repellent and DAT, as the repellents were less effective at 20 than at 10 and 30 DAT (Table 1, Fig. 4). Captures of beetles per trap placed far from the treated trees were not significantly different from captures in control plots for either push (repellent alone) or combined push-pull treatments (Table 1, Fig. 4).

**Table 1:** Results from the generalized linear model with Poisson distribution. Two separate models were run for traps deployed close (~5–10 m) and far (~1–1.5 m) from dispensers releasing repellent treatment.

Parameter	Df	Χ^2^	P value
**Close traps**			
Push (repellents)	2	28.093	<0.001
Pull (ethanol)	1	1.433	0.231
Sampling date	2	0.1154	0.944
Push × pull	2	13.458	0.001
Push × sampling date	4	17.652	0.001
Pull × Sampling date	2	0.5575	0.757
**Far traps**			
Push (repellents)	2	8.299	0.348
Pull (ethanol)	1	24.397	0.013
Sampling date	2	51.202	0.001
Push × pull	2	14.374	0.161
Push × sampling date	4	16.704	0.373
Pull × Sampling date	2	14.212	0.164

### Ambrosia beetle diversity and community composition

**Figure 5:**
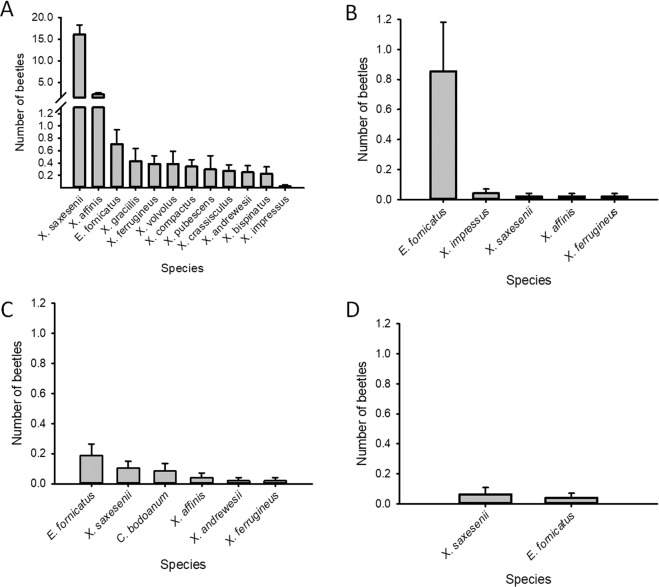
Diversity and quantity of ambrosia beetles collected by associated semiochemical treatment. Mean (±SEM) of ambrosia beetles captured, per species. (**A**) ethanol lures, (**B**) untreated control, (**C**) plots treated with methyl salicylate SPLAT (far and close traps combined), (**D**) plots treated with verbenone SPLAT (far and close traps combined). ‘Close’ indicates monitoring traps were ~5–10 cm from repellent dispenser, while ‘far’ indicates traps were ~1–1.5 m from repellent. Note the change in the scales of the y axis with reference to the reduction of beetles observed with MeSA and verbenone treatments. Genus names by abbreviation: A. = *Acropteroxys*, C. = *Cychlorhipidion*, D. = *Dryocoetoides*, E. = *Euwallacea*, H. = *Hylocurus*, and X. = *Xyleborus*.

Sixteen species of scolytine beetles were captured during the course of the experiment (Table 2). The vector of *R. lauricola* in redbay *X. galbratus* was not found during either field study. However, five species known to carry and for some of them transmit *R. lauricola* were found: *Xyleborus ferrugineus, Xyleborus gracilis, X. bispinatus, X. volvulus*, and *Xyleborus crassisculus*. Trees with the ethanol pull and untreated trees were both dominated by a single species [*X. saxesenii* 75% (Fig. 5A) and *E. fornicatus* 85% (Fig. 5B), respectively]. The community composition of beetles was different on traps placed in the repellent blocks from the composition on the traps placed with ethanol lures; verbenone associated traps only captured three species of beetles while the methyl salicylate and the untreated control captured seven or more species (Fig. 5D). The most species of xyleborine ambrosia beetles were captured on traps associated with the ethanol lure (Fig. 5A). One species was only found on traps in plots treated with 10% w/v methyl salicylate released from SPLAT: *Cyclorhipidion bodoanum*; but in very low numbers (Table 1, Fig. 5C). Capture of no species increased in the presence of verbenone; *E. fornicatus* were captured in higher numbers on traps in untreated control traps than on traps deployed in any other treatment plots.

**Table 2:** Scolytinae beetles collected in avocado grove affected by laurel wilt, summer 2018.

Species name	Sum	*R. lauricola* carrier	References
*Xyleborinus saxesenii (*Ratzeburg)	744	Low^a^	^7,38^
*Xyleborus affinis (*Eichhoff)	104	Low^a^	^7,38^
*Euwallacea* nr. *Fornicates* (Eichhoff)	83	Unknown	
*Xyleborus ferrugineus* (Fabricius)	19	Yes	^7,38^
*Xyleborus gracilis* (Eichhoff)	19	Yes^b^	^7,38^
*Xyleborus volvulus* (Fabricius)	19	Yes	^7,38^
*Xylosandrus compactus* (Eichhoff)	15	No	^38^
*Xyleborus pubescens* (Zimmermann)	13	Unknown	
*Xylosandrus crassisculus* (Motschulsky)	12	Yes^a^	^7,38^
*Xyleborinus andrewesii* (Ratzeburg)	12	Unknown	
*Xyleborus bispinatus* (Eichhoff)	10	Yes	^7,38^
*Cyclorhipidion bodoanum* (Reitter)	4	Unknown	
*Xyleborus impressus* (Eichoff)	3	Unknown	
*Hylocurus flaglerensis* (Blackman)	2	Unknown	
*Ambrosiodmus lecontei* (Hopkins)	2	Low^c^	^38^, Simmons *et al*. 2016

Sum: total number of individuals collected during push-pull field trial.

^a^Transmission assays have been conducted by^7^ on these species, and even if these species carried the pathogen they were not able to infect avocado.

^b^*X. gracilis* has been reported to be able to transmit *Raffaelea lauricola* to avocado by^7^; however^38^, did not found any individual carrying the pathogen after examination of field collected individuals.

^c^*R. lauricola* was not found in *A. leconti* by^38^, but found by Simmons *et al*. (2016).

**Figure 6:**
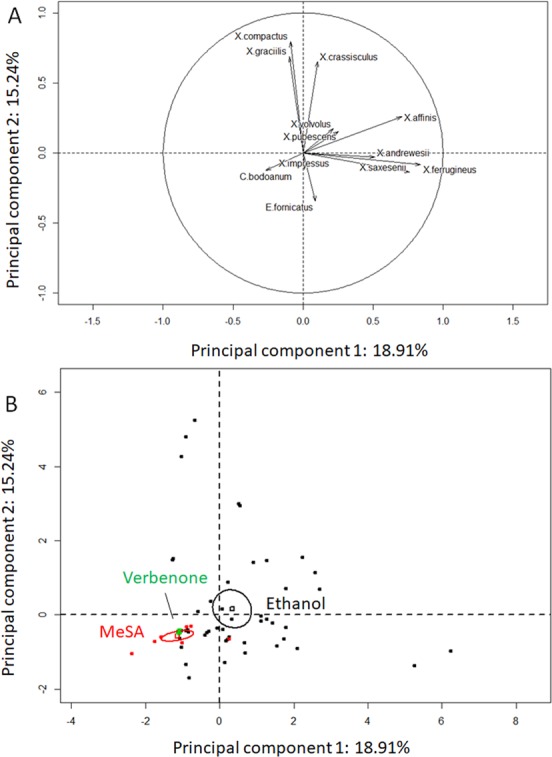
Principal component analysis of ambrosia beetle (Coleoptera: Curculionidae: Scolytinae and Platypodinae) species collected in avocado orchards in presence of push-pull treatments. (**A**) Contributions of beetle species on the first and second principal components. (**B**) Confidence ellipses highlighting the effects of ethanol lures, and methyl salicylate (MeSA) and verbenone on beetle catch on monitoring traps.

A principal component analysis was performed to determine the main contributions to the differences in beetle diversity associated with various semiochemical treatments. The first (PC1) and second (PC2) principal components accounted for 18.91 and 15.24% of the variance, respectively and the other principal components explained less than 10% of the variance. PC1 indicated positive correlation between multiple species and the ethanol lures [*X. saxesenii* (t = 10.941, P < 0.001), *X. affinis* (t = 6.063, P < 0.001), and *X. ferrugineus* (t = 11.360, P < 0.001)] (Fig. 6A). PC2 was negatively correlated with *E. fornicatus* (t = 2.767, P = 0.007), and positively correlated with *X. crassisculus* (t = 7.094, P = <0.001), *X. compactus* (t = 7.921, P = <0.001), and *X. graciliis* (t = 5.792, P = <0.001) (Fig. 6A). Overall, there was a clear separation in the species associated with the ethanol lures and the repellents with larger diversity associated with the repellents (Fig. 6B). Among the beetle species captured, *X. affinis*, and *X. saxensenii* were most attracted by ethanol lures.

## Discussion

Redbay ambrosia beetles, *X. glabratus*, and laurel wilt have decimated redbay trees in the southeastern US^36^. Laurel wilt has also severely impacted the avocado industry in southern Florida. Redbay ambrosia beetles are not the main vector(s) of *R. lauricola*, the causal fungus of laurel wilt in commercially grown avocado. Previous investigations indicate that avocado is among the least attractive of the Lauraecae to *X. glabratus*^28^. Therefore, *X. glabratus* rarely occurs in avocado orchards^22^, which was further confirmed by the current results; no *X. glabratus* were captured during both field experiments. The transmission of *R. lauricola* to avocado is likely the result of attack by a diversity of ambrosia beetles that acquire the pathogenic fungi by horizontal transfer in infected Lauracea (redbay or swamp bay)^7^. In Florida, avocado groves are located nearby the Everglades National Park that has been severely impacted by laurel wilt^37^. It is possible that the Everglades, as well as, small urban forest patches serve as a source of infected ambrosia beetles that migrate subsequently in avocado. Herein, we found 16 species of ambrosia beetles, with some (*X. crassisculus, X. bispinosa* and *X. gracilis*) confirmed vectors of *R. lauricola*^7^. Furthermore, Ploetz *et al*.^38^ reported that most ambrosia beetles harbor viable *R. lauricola* spores. This suggests that control of laurel wilt in avocado should not focus on a single species, but on the community of ambrosia beetles occurring in the crop when *R. lauricola* is present or nearby.

Traps placed with ethanol lures captured the highest diversity of xyleborine ambrosia beetles (Fig. 5A). In the field, *X. volvulus* was only found on traps in plots treated with the low-dose ethanol lures (pull) (Fig. 5A). *E. fornicatus* and *X. saxesnii* were captured on traps placed in all treatment plots. Trap captures of *X. ferrugineus, X. saxesnii*, and *X. affinis* were higher in plots treated with ethanol lures than in all plots without the pull treatment. Furthermore, the MeSA treatment slightly increased capture of three species compared with captures in control plots indicating that MeSA may also act as attractant for certain ambrosia beetle species^5^. Our results suggest that management of a complex of Scolytineae beetles in avocado with semiochemical and in particular the xyleborine beetles, would likely require use of a pull treatment (ethanol lures) in addition to some general repellent treatment. Variation among host plant preference between ambrosia beetle species may explain the variation observed in the communities of beetle species found in avocado and their contribution to disease incidence.

With the high risk of laurel wilt to avocado orchards in southern Florida, it is essential to develop tools for prevention and management of immigrating ambrosia beetles. It is unclear which specific species contribute most to disease spread in avocado or their proportional contribution as vectors^7,9^. With the threat of lateral pathogen spread between various species, it is a conservative approach to assume all species are potentially capable of promoting pathogen spread. The push-pull strategy of applying combined repellent and attractant stimuli in order to manipulate the distribution of a pest is an integrated pest management tool that has been established in several systems^39^. The use of repellents has been successful in natural stands of Laureacae against laurel wilt^27^, but in a monoculture, agricultural setting, we hypothesized that the additional pull treatment may be necessary to affect beetle populations based on our first trial where repellents alone in avocado were ineffective.

Under laboratory conditions, we assessed the repellency of verbenone and methyl salicylate on two putative vectors of *R. lauricola*: *X. bispinatus* and *X. volvulus*. Verbenone was selected because it has been used in multiple systems as a general bark beetle repellent and has also been proven highly effective in natural forests against the redbay ambrosia beetle, *X. glabratus*^27,40–44^. Methyl salicylate, the other putative repellent used in our studies, is a volatile chemical commonly associated with plant stressors^45^. It may or may not be attractive or repellent to beetle species depending on their life history strategy^5^. MeSA was found to be repellent to *X. glabratus* in laboratory studies^34^, and confirmed later under field conditions^27^. This is congruent with the life history strategy of *X. glabratus*, which is known to attack healthy trees^20^. However, its behavioral effect on other Ambrosia beetle species is unclear. It could potentially be an attractant for secondary and tertiary colonizers and a repellent for others.

The laboratory studies did not provide a clear connection to the field studies. In our laboratory studies, methyl salicylate did not appear to be a broad-spectrum repellent of Ambrosia beetles species with low-level repellency to *X. bispinatus*, but not to *X. volvulus*. In our field studies, *X. bispinatus* and *X. volvulus* were not found in the verbenone treatment but *X. volvulus* was found in the methyl salicylate treatment which validates the lab data showing it is not a repellent for this species. *X. bispinatus* was only found in the low-dose ethanol baited traps but in very low numbers. While laboratory olfactometer assays can be useful for preliminary screening, they may not effectively predict what is to occur in the field because of the potential effects of uncontrolled variables such as presence of host and non-odors from surrounding plants, varying levels of disease, and a heterospecific community of beetles. This is particularly the case with verbenone, that did not statistically repel either *X. bispinatus* or *X. volvulus* in the laboratory, but was effective in repelling most of the beetles present in commercial avocado, despite the limited radius of activity from dispensers tested here.

Information gathered from the first field study guided the design of second experiment. Beetle captures in the first study without the ethanol lures varied over time following treatment application. This variation seemed to be due to natural population fluctuations of beetles. However, at 20 DAT when the majority of beetles were captured, there was also an effect of treatment. Thus, it was prudent to hypothesize that effectiveness of a repellent treatment may vary in proportion to fluctuations in beetle population density when planning the second experiment. The results of the second experiment incorporating the pull treatment (ethanol lure) appeared to confirm this hypothesis. The synergy between the push and pull treatments was observed as significant reductions in beetle captures relative to control plots at 10 and 30 DAT, but not at 20 DAT, when the density of beetles was lower than during the other sampling dates. Catches of beetles were reduced most effectively relative to control plots when combining the forces of push and pull as a treatment. However, this was only evident on traps placed “close” (~5–10 cm) to verbenone dispensers indicating a small effective repellent radius [*sensu* effective attraction radius (EAR) measure^46^] of this treatment.

The effective repellent radius of the verbenone-releasing dispensers tested here was less than 1 meter. Thus, there would need to be a high density of these dispensers for practical application. Because of the small effective radius, SPLAT loaded with the 10% w/v rate of verbenone would likely need to be applied to every tree within a monoculture of avocado and in combination with low-dose ethanol lures on field edges. In the present study, the treatments were effective up to 30 DAT, which is consistent with the upper limit of longevity observed previously (Martini, unpublished data).

The field site was selected because it was highly managed with fungicides and had no active disease symptoms, but was in close proximity to a low-management site with active laurel wilt. The flight capability of *X. glabratus* and another ambrosia beetle species, *Monarthrum mali* Wood & Bright, have been assessed and indicate that ambrosia beetle may potentially immigrate from distances up to 100 m^47^. An edge is a boundary between two distinct habitats or vegetation types or in this case, two management styles. The effect of edges has been long studied in other contexts such as wildlife conservation^48^. Thus, a follow-up study is warranted to investigate the edge effect to determine the distance immigrating beetles penetrate into avocado orchards in order to best determine where and how semiochemical dispensers and sentinel traps should be placed.

Management is crucial to prevention of the spread of laurel wilt within avocado orchards. Our results suggest that the push-pull strategy may hold promise as a management tool for this pathosystem and deserves further investigation. It is unlikely that it could adequately manage beetle populations as a stand-alone treatment and would likely function best in association with additional preventive measures such as removal of infected trees. Several follow up investigations are warranted. It is possible that long-term effects of beetle population suppression with push-pull could reduce disease incidence over time, if semiochemicals were deployed on an area-wide scale (see Conlong *et al*.)^49^. This could be evaluated by tracking infections in high-risk Lauraceous hosts occurring nearby commercial avocado^19^ in addition to infection sentinels monitored within treated avocado^50^. If fully refined, a push-pull approach would benefit from a complementary integrated pest management program against laurel wilt that includes removal and destruction of infected trees. Furthermore, the economic viability of this potential tactic with and without fungicide treatments deserves further investigation.

## Supplementary information

Supplementary information

## Data Availability

The data and SAS/R scripts related to this study are available from the corresponding author upon request.
