# Supplementary material for: Evaluation of semiochemical based push-pull strategy for population suppression of ambrosia beetle vectors of laurel wilt disease in avocado
Source: Sci Rep. 2020 Feb 14;10:2670. doi: 10.1038/s41598-020-59569-0 (PMC7021720; doi:10.1038/s41598-020-59569-0)
Supplement: Supplementary file 1 — Supplementary information [file 41598_2020_59569_MOESM1_ESM.pdf]

Evaluation of semiochemical based push-pull strategy for population suppression of ambrosia beetle vectors of laurel wilt disease in avocado

Monique J. Rivera[1,2,7], Xavier Martini[1,3], Derrick Conover[3], Agenor Mafra-Neto[4], Daniel Carrillo[5], and Lukasz L. Stelinski[6]

1 Authors had equal contribution to this paper

2 Department of Entomology, University of California Riverside, Riverside, CA, USA

3 Department of Entomology and Nematology, North Florida Research and Education Center, University of Florida, Quincy, FL, USA

4 ISCA Technologies, Inc., Riverside, CA, USA

5 Department of Entomology, Tropical Research & Education Center, University of Florida, Homestead, FL, USA

6 Department of Entomology and Nematology, Citrus Research and Education Center, University of Florida, Lake Alfred, FL, USA

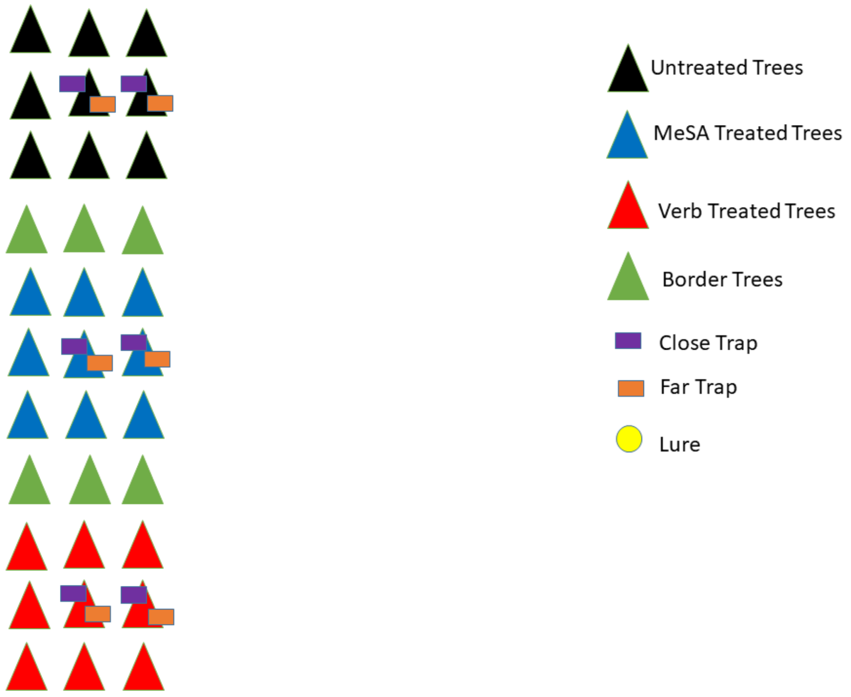

S1: This figure represents a single block of treatments for the first field trial. There are no lures present in this trial but SPLAT and two associated sticky traps are shown on two trees within each block. Traps were placed ‘close’ (~ 5-10 cm) to the SPLAT dispensers and ‘far’ (~1-1.5 m) from dispensers within the same tree.

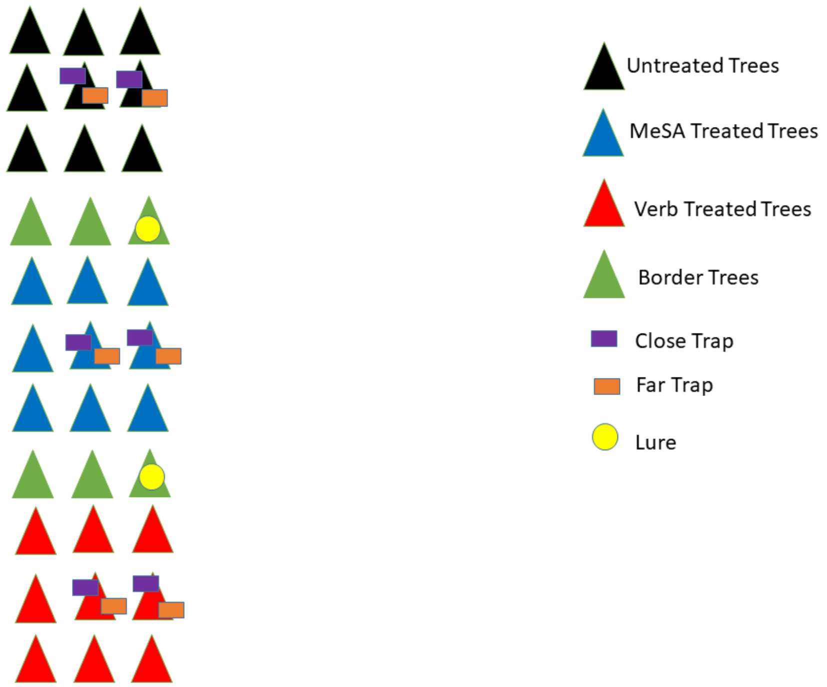

S2: This figure represents a single block of treatments for the second field trial. There are lures present on the buffer rows in this trial with SPLAT applied to the treatment blocks and as in the previous trial, two associated sticky traps are shown on two trees within each block. Traps were placed ‘close’ (~ 5-10 cm) to the SPLAT dispensers and ‘far’ (~1-1.5 m) from dispensers within the same tree.
